# β-Carboxysome bioinformatics: identification and evolution of new bacterial microcompartment protein gene classes and core locus constraints

**DOI:** 10.1093/jxb/erx115

**Published:** 2017-04-17

**Authors:** Manuel Sommer, Fei Cai, Matthew Melnicki, Cheryl A Kerfeld

**Affiliations:** 1 Department of Plant and Microbial Biology, UC Berkeley, Berkeley, CA, USA; 2 MSU-DOE Plant Research Laboratory and Department of Biochemistry and Molecular Biology, Michigan State University, East Lansing, MI, USA; 3 MBIB Division, Lawrence Berkeley National Laboratory, Berkeley, CA, USA

**Keywords:** Bacterial microcompartment, β-carboxysome, BMC, CcmK, CcmO, cyanobacteria, evolution, phylogeny, regulation

## Abstract

Carboxysomes are bacterial microcompartments (BMCs) that enhance CO_2_ fixation in all cyanobacteria. Structurally, carboxysome shell proteins are classified according to the type of oligomer formed: hexameric (BMC-H), trimeric (BMC-T) and pentameric (BMC-P) proteins. To understand the forces driving the evolution of the carboxysome shell, we conducted a bioinformatic study of genes encoding β-carboxysome shell proteins, taking advantage of the recent large increase in sequenced cyanobacterial genomes. In addition to the four well-established BMC-H (CcmK1–4) classes, our analysis reveals two new CcmK classes, which we name CcmK5 and CcmK6. CcmK5 is phylogenetically closest to CcmK3 and CcmK4, and the *ccmK5* gene is found only in genomes lacking *ccmK3* and *ccmk4* genes. *ccmK6* is found predominantly in heterocyst-forming cyanobacteria. The gene encoding the BMC-T homolog CcmO is associated with the main carboxysome locus (MCL) in only 60% of all species. We find five evolutionary origins of separation of *ccmO* from the MCL. Transcriptome analysis demonstrates that satellite *ccmO* genes, in contrast to MCL-associated *ccmO* genes, are never co-regulated with other MCL genes. The dispersal of carboxysome shell genes across the genome allows for distinct regulation of their expression, perhaps in response to changes in environmental conditions.

## Teaser

Analysis of β-cyanobacteria genomes reveals new carboxysome shell protein (CcmK) classes and demonstrates that relocation of shell protein genes to satellite loci increases regulatory plasticity of this microcompartment shell.

## Introduction

The formation of subcellular compartments is fundamental to eukaryotic life, but it is now well accepted that bacteria, too, can form metabolically active organelles (recently reviewed in Kerfeld and Erbilgin, 2015). The observation of polyhedral bodies in cyanobacteria in 1956 marks the discovery of a group of organelles termed bacterial microcompartments (BMCs) (Niklowitz and Drews, 1956). These cyanobacterial BMCs were shown to contain ribulose-1,5-bisphosphate carboxylase/oxygenase (RubisCO) and were thus termed carboxysomes (Shively *et al.*, 1973). Based on sequence homology to carboxysome shell proteins, BMCs were also detected in heterotrophic bacteria (Stojiljkovic *et al.*, 1995; Kofoid *et al.*, 1999; Bobik *et al.*, 1999) and are now known to be present in at least 23 bacterial phyla (Axen *et al.*, 2014). While the encapsulated proteins of different BMC types catalyse diverse chemical reactions (Axen *et al.*, 2014; Kerfeld and Erbilgin, 2015), a common feature to all BMCs is the proteinaceous BMC shell. Structural studies revealed three distinct types of BMC shell proteins: BMC-H, a hexamer-forming protein that contains a single Pfam00936 domain (Kerfeld *et al.*, 2005); BMC-T, a trimer-forming protein that contains two consecutive Pfam00936 domains (Klein *et al.*, 2009; Cai *et al.*, 2013); and BMC-P, a pentamer-forming protein that contains a single Pfam03319 domain (Tanaka *et al.*, 2008; Sutter *et al.*, 2013). The carboxysome shell has been modeled as an icosahedron, with the BMC-H and BMC-T proteins forming the facets, and the BMC-P proteins forming the vertices (Tanaka *et al.*, 2008). Construction of an icosahedral shell requires only one type of hexameric and one type of pentameric protein; however, all BMC-containing bacteria encode for multiple copies of highly similar BMC-H or BMC-T proteins that are usually collectively located in a single BMC operon, and there may be satellite loci containing additional shell proteins (Axen *et al.*, 2014).

Carboxysomes are BMCs, 90 to 600 nm in diameter, that are found in all cyanobacteria, and some chemoautotrophic and purple bacteria. (Cannon *et al.*, 2001; Badger and Price, 2003; Tanaka *et al.*, 2008; Axen *et al.*, 2014; Gonzalez-Esquer *et al.*, 2016*b*). Besides RubisCO, carboxysomes also encapsulate one or more carbonic anhydrases that catalyse the conversion of the negatively charged bicarbonate to CO_2_ (Price *et al.*, 1992; Heinhorst *et al.*, 2006; Sawaya *et al.*, 2006; Cannon *et al.*, 2010; Peña *et al.*, 2010; McGurn *et al.*, 2016). Thus, CO_2_ is enriched in the vicinity of RubisCO and its leakage is prevented by the impermeability of the carboxysome shell to CO_2_ (Dou *et al.*, 2008). Selective permeability of the carboxysome shell is hypothesized to be achieved by pores in the center of each shell protein oligomer, which are 3.5–7 Å wide in BMC-H and BMC-P proteins (Kerfeld *et al.*, 2005; Tanaka *et al.*, 2008; Kinney *et al.*, 2011; Sutter *et al.*, 2013), and 13–14 Å wide in BMC-Ts (Klein *et al.*, 2009; Cai *et al.*, 2013). The central pore of a BMC shell protein oligomer is encircled by charged amino acid sidechains, which is assumed to promote the passage of polar and charged metabolites (Kerfeld *et al.*, 2005).

There are two known types of carboxysomes that are distinguished based on the form of RubisCO they encapsulate, Form IA in α-carboxysomes and Form IB in β-carboxysomes (Tabita, 1999; Badger and Bek, 2008). Different types of carbonic anhydrases are utilized for the production of CO_2_ in the carboxysome lumen, namely CcmM and CcaA in β- and CsoSCA in α-carboxysomes. Furthermore, a protein involved in structural assembly of carboxysomes, CcmN in β- and CsoS2 in α-carboxysomes, is encoded in the respective MCL (Kinney *et al.*, 2012; Cai *et al.*, 2015; Chaijarasphong *et al.*, 2016); CsoS2 and CcmN share no sequence homology (Cannon *et al.*, 2002; Badger *et al.*, 2002; Badger and Price, 2003; Zarzycki *et al.*, 2013; Kerfeld and Melnicki, 2016). The MCL of α-cyanobacteria contains nearly all shell components, only the BMC-T-encoding *csoS1D* gene is separated from this locus by a single gene (Roberts *et al.*, 2012). In contrast, some components of the β-carboxysome are encoded in a varying number of ‘satellite loci’ that are distant from the MCL (Axen *et al.*, 2014).

A typical β-cyanobacterial genome contains four BMC-H genes, termed *ccmK1–4*, two of which are located in a satellite locus and the other two are expressed in the MCL (Kinney *et al.*, 2011). However, the number of *ccmK* copies in the MCL may vary between one and three (Rae *et al.*, 2013). Deletion of the MCL-encoded *ccmK2* gene in *Synechococcus elongatus* PCC 7942 (Syn7942) causes a carboxysome-less phenotype, underscoring its importance to the formation of the carboxysome shell (Price *et al.*, 1993; Rae *et al.*, 2012; Cameron *et al.*, 2013). Deletion of *ccmK* genes located in a satellite locus of the Syn7942 genome, *ccmK3* and *ccmK4*, causes a growth retarded phenotype with apparently intact carboxysome shells, while either single deletion mutants grow like wild type cells (Rae *et al.*, 2012). It has thus been hypothesized that CcmK3 and CcmK4 are functionally redundant proteins that play only a minor structural role (Rae *et al.*, 2012). The BMC-T gene, *ccmP*, forms another satellite locus in β-carboxysomes. The second BMC-T gene, *ccmO*, is positionally variable, as it can be either part of the MCL or a satellite gene (Rae *et al.*, 2013; Axen *et al.*, 2014). Since its gene deletion causes complete lack of carboxysomes, a major structural role was proposed for CcmO (Marco *et al.*, 1994; Rae *et al.*, 2012; Cameron *et al.*, 2013), but the CcmO protein has proven recalcitrant to crystallization and its structure remains unknown.

The genomic location of carboxysome genes has been addressed by a number of comparative studies (Kinney *et al.*, 2011; Rae *et al.*, 2013; Axen *et al.*, 2014). When cyanobacterial α-carboxysomes were first purified, carboxysome loci from multiple *Prochlorococcus* species were comprehensively analysed to identify new putative carboxysome genes (Roberts *et al.*, 2012). However, no similar analysis of main and satellite loci encoding β-carboxysomes has been published to date. In 2013, the number of sequenced β-cyanobacterial genomes more than doubled in a single study aimed to provide a more ecophysiologically representative coverage of the cyanobacterial phylum (Shih *et al.*, 2013). Since then, the number of available cyanobacterial genomes has increased to 395. Using these data we undertook a comprehensive bioinformatic analysis of β-carboxysome loci to analyse their genome location and content with regard to their proposed function and to reveal driving forces of genome evolution on β-carboxysome genes.

## Material and methods

### Building a database of CcmK homologs from β-carboxysomes

In order to accurately assign annotations, a hidden Markov model (HMM) was built for each of CcmK1/2, CcmK3, CcmK4, CcmO, CsoS1A/B/C, CsoS1D, CsoS1E and EutM from alignments of seven to 29 homologs of the respective protein class from finished and annotated genomes after manual curation. Amino acid sequences from proteins with an assigned BMC (Pfam00936) domain in all cyanobacterial genomes were collected from the Joint Genome Institute (JGI) Integrated Microbial Genomes/Metagenomes Expert Review (IMG/MER) database (Markowitz *et al.*, 2012) on 2 August 2016. Each sequence from this collection was aligned and scored to all eight HMMs using the HMMer suite (www.hmmer.org, version 3.0) to assign preliminary annotation based on the lowest scored e-value. Species were annotated as α- or β-cyanobacteria according to whether they contained a carboxysome locus with *cso* or *ccm* type genes, respectively. Species with no *ccm* genes were classified as ‘unknown’. Proteins from α- or unknown type cyanobacteria, as well as proteins that were annotated as CcmO, were removed for the subsequent phylogenetic analysis. The remaining 955 proteins were manually curated by deleting pseudogenes, a gene with low information content (>20% ‘X’) and genes from seven incompletely sequenced genomes, leaving 938 proteins from 227 genomes in the final dataset.

### Phylogenetic analysis of CcmK homologs from β-carboxysomes

EutM from *Escherichia coli* UMNF18 and PduA from *Salmonella enterica enterica* sv. Typhimurium UK-1 were downloaded from IMG/MER and added to the collection as an outgroup and the sequences were aligned using the L-INS-I algorithm of the MAFFT suite (Markowitz *et al.*, 2012; Katoh and Standley, 2013). Redundancy was reduced by removing sequences with >99% identity using jalview software (Waterhouse *et al.*, 2009). Columns with low information content were subsequently removed from the alignment using the automated algorithm from TrimAL (Capella-Gutiérrez *et al.*, 2009). The phylogram was created with PhyML, using the Le–Gascuel (LG) substitution model, nearest neighbor interchange (NNI) type of tree improvement and the approximate likelihood ratio test (aLRT) Shimodaira–Hasegawa (SH)-like method to calculate branch support values (Guindon *et al.*, 2010). The phylogram was labelled, rooted and colored in Archaeopterix (Han and Zmasek, 2009). To assign proteins in the mixed CcmK1/2 cluster to the CcmK1 or CcmK2 class, protein lengths were plotted in a histogram. Shorter proteins (100–107 aa) were named CcmK2 and longer proteins (108–118 aa) were named CcmK1.

### Conservation study of CcmK residues

Multiple sequence alignments of subsets of the dataset above were separately created for CcmK1/2 and CcmK3–6. Conservation scores were calculated with the Consurf online tool (Ashkenazy *et al.*, 2010), and were visualized using Protein Data Bank (pdb) files in the magenta_white_cyan color gradient to display decreasing conservation in pymol (The PyMOL Molecular Graphics System, Version 1.8, Schrödinger, LLC). The pdb entries used as scaffolds on which conservation scores were plotted included 3bn4 (for CcmK1 and the homology model of *Nostoc* sp. PCC 7524 CcmK6), 3ssr (for CcmK2 and the homology model of *Cyanothece* sp. PCC7425 CcmK5) and 2a18 (for CcmK4 and the homology model of Syn7942 CcmK3) (Kerfeld *et al.*, 2005; Tanaka *et al.*, 2008; Samborska and Kimber, 2012). Homology models were built using the SWISS-MODEL online tool (Biasini *et al.*, 2014). HMM logos for all classes were created using the Skylign online tool with standard settings (observed counts used, full length alignment, letters contain all information) (Wheeler *et al.*, 2014). HMM logos were manually aligned for comparison.

### Calculation of electrostatic surface potential

Electrostatic potentials were calculated for the structural models used in the conservation study. The surface potential was calculated with APBS toolkit v2 in PyMOL and potentials between –10 and 10 kT/e were visualized with a color gradient from red (negative) over white (neutral) to blue (positive) (Baker *et al.*, 2001).

### Co-occurrence study of CcmK proteins

CcmK protein co-occurrence was visualized in a Venn diagram. Circles in the diagram were sized based on the number of genomes they represent with no linear correlation between area and genome number. To determine unexpected frequencies of coexistence of CcmK gene pairs, numbers of expected co-occurences were calculated from the product of the relative genome coverage of both CcmK proteins multiplied by the number of genomes analysed (227). The ratio of observed and expected co-occurrences was calculated to display positive (values >1) or negative (values <1) deviation of the expected correlation. To test statistically significant deviation from the expected number of co-occurrences, a chi-squared test was performed on each pair of CcmKs (*P<0.05, **P<0.01 and ***P<0.001).

### Comparative analysis of main and satellite loci encoding β-carboxysome shell genes

To compare the structure of the main carboxysome loci across species, positional information for genes with Pfam00936 domains were extracted. To gather sequence and positional information for the remaining MCL genes (*ccmL*, *ccmM*, and *ccmN*) and the satellite gene *ccmP*, the representative Syn7942 protein sequence was compared with all protein sequences in the JGI IMG/MER database using BLASTP. The e-value cutoff was 10^–50^ for CcmP and CcmM, and 10^–5^ for CcmL and CcmN. To test for completeness of the additional datasets, five more BLAST searches with randomly picked hits from the initial search using the same cutoff were performed. No additional proteins were detected in these control runs. Locus assembly was based on adjacency of carboxysome gene IDs. Insertions of single non-carboxysome genes was not allowed to interrupt recognition of the MCL. Genes were recognized as satellites only if there was no other carboxysome gene within 10 open reading frames. For locus analysis, identical types of loci were grouped and counted; subsequently, representative schematics were drawn. To visualize the phylogenetic distribution of relocated *ccmO* genes, a phylogram of all cyanobacterial genomes (a species tree) was annotated, recolored and replotted. A phylogram of all CcmO proteins in the sequence collection was generated as described for CcmK homologs.

### Correlation of carboxysome gene expression

Ten published transcriptome datasets from eight different cyanobacterial species, with data from at least three different conditions each, were analysed (Stöckel *et al.*, 2008; Flaherty *et al.*, 2011; Schwarz *et al.*, 2011; Straub *et al.*, 2011; Hernandez-Prieto and Futschik, 2012; Ludwig and Bryant, 2012*a*; Park *et al.*, 2013; Billis *et al.*, 2014; Panyakampol *et al.*, 2015; Yingping *et al.*, 2015). Six of these studies included species in which CcmO is encoded in the MCL; the other four species encoded CcmO in a satellite locus. Where datasets contained log2 transformed data, data were linearized. Expression data for *ccmK1–4*, *ccmL*, *ccmM*, *ccmN*, *ccmO* and *ccmP* were extracted and the pairwise Pearson correlation was calculated, followed by calculation of the mean correlation of selected genes with MCL genes. For *ccmO*, the mean correlation with MCL *ccmKs*, *ccmM*, *ccmN*, and *ccmL* was calculated for each species. For *ccmK1*/*2* and *ccmK3*/*4*, the determined correlation value was the averaged mean correlation with all remaining MCL genes from the respective species, except RubisCO genes.

## Results

### Phylogenetic analysis of CcmK homologs reveals at least two new CcmK classes

**Fig. 1.**
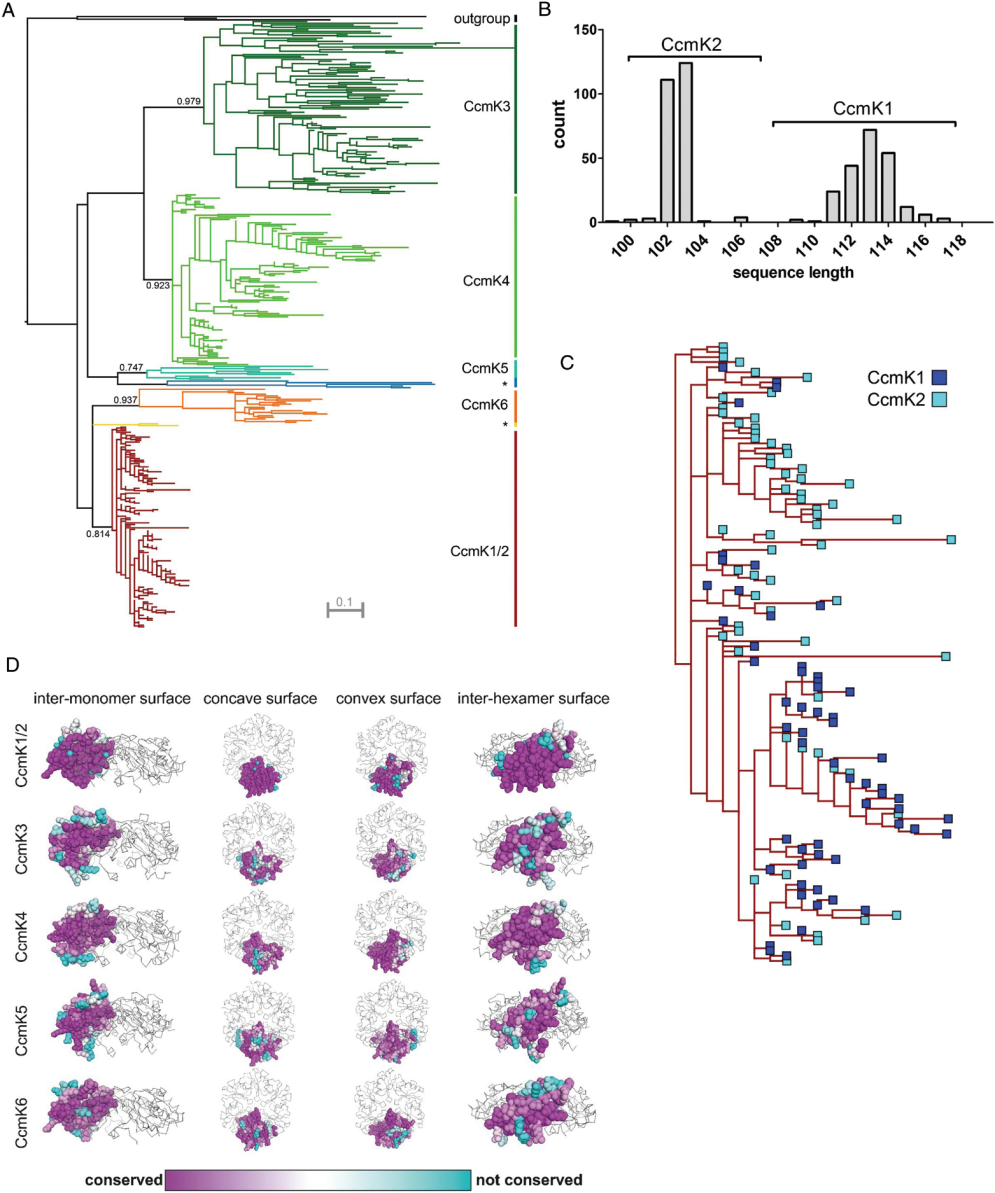
(A) Phylogram of CcmK proteins from 227 β-cyanobacterial genomes with less than 99% sequence identity. Numbers show branch support values. Classes labelled with asterisks are subclasses of CcmK5 and CcmK6, CcmK5* and CcmK6*, respectively. (B) Histogram of CcmK genes in the main β-carboxysome locus based on sequence length. Brackets indicate borders of gene annotation for subsequent analysis. (C) Enlarged view on the CcmK1/2 superclass from 1A. (D) Residue-based conservation score plotted for structures (CcmK1/2, CcmK4) or homology models (CcmK3, CcmK5, CcmK6) of hexamer-forming shell proteins. Grey ribbons indicate position of remaining monomers within each hexamer complex.

To classify the CcmK proteins from all sequenced cyanobacteria genomes, a phylogram of non-redundant BMC-H proteins from species with β-carboxysomes was built (Fig. 1A). The phylogram displayed three distinct branches for the most common classes of CcmK proteins. One branch contained all CcmK1 and CcmK2 proteins, and the other two contained all CcmK3s and all CcmK4s, respectively. In addition to these previously defined classes, at least two new classes of CcmK proteins were apparent, appearing as distinct clusters on the phylogram (Fig. 1A). The two new classes were named CcmK5 and CcmK6; a long sub-branch that was formed by a few proteins within each new class was named CcmK5* and CcmK6*, respectively. Branch support values for CcmK1–4 ranged from 0.81 to 0.98. The branch support was not significantly different in the new classes, being 0.75 in CcmK5 and 0.94 in CcmK6. CcmK1 and -2 proteins clustered together in a mixed class, and within the CcmK1/2 cluster, proteins of both classes could not be phylogenetically separated after a sequence length-based annotation (Fig. 1B, C). The mixed CcmK1/2 class, on average, showed short branches, which reflected that the amino acid substitution rate was low between homologs. Very few of these amino acid substitutions in the CcmK1/2 class were found at the inter-monomer or inter-hexamer interfaces (Fig. 1D). In contrast, branches of the CcmK3 and CcmK4 proteins were much longer and residues at the inter-monomer contacts were less conserved, especially in CcmK3. The branch length in CcmK5 was more similar to CcmK3 and -4, while CcmK6 was more similar to CcmK1 and -2. The suggested evolutionary path of the phylogram similarly positioned CcmK5 closer to CcmK3 and -4, whereas CcmK6 was closer to CcmK1 and -2.

**Fig. 2.**
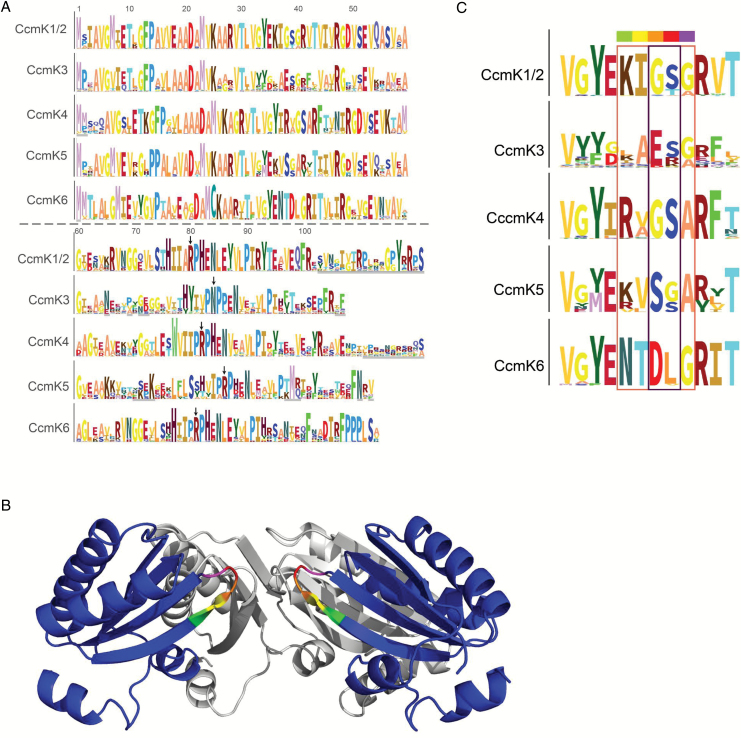
(A) HMM logos of all CcmK subtypes; numbers indicate residue number based on CcmK1/2 HMM logo. Gray scalebars on *y*-axis of each logo correspond to 6.45 bits conservation. Residues with <90% coverage are underlined light gray. A specific color was arbitrarily assigned to each residue to increase legibility. Black arrows highlight the residue corresponding to R79 from PduA. (B) Side view of two opposing monomers in a CcmK4 hexamer (based on pdb entry 2a18; dark blue). The pore residues are rainbow colored. (C) Enlarged HMM logos of pore residues in all CcmK classes. Rainbow color-code matches (B).

Hidden Markov model (HMM) logos of the six CcmK classes were built to compare frequency distributions of characteristic BMC residues in the new CcmK classes (Fig. 2A). Highly conserved residues of the CcmK1–4 HMMs were also found conserved in the newly identified classes, CcmK5 and CcmK6. The HMM logos for each of the CcmK classes were aligned to compare residues that presumably flank the pore (Fig. 2B, C). The residues predicted to surround the pore in CcmK5 and CcmK6 hexamers were highly conserved but distinct from other CcmK classes. Residues predicted to surround the pore in a CcmK5 hexamer were similar to those of CcmK1/2 and CcmK4, but the central pair of a glycine and a serine residue (color-coded orange and red) at the most constricted position in the pore was swapped. The residues predicted to converge around the CcmK6 hexamer pore include an acidic residue as in CcmK3.

**Fig. 3.**
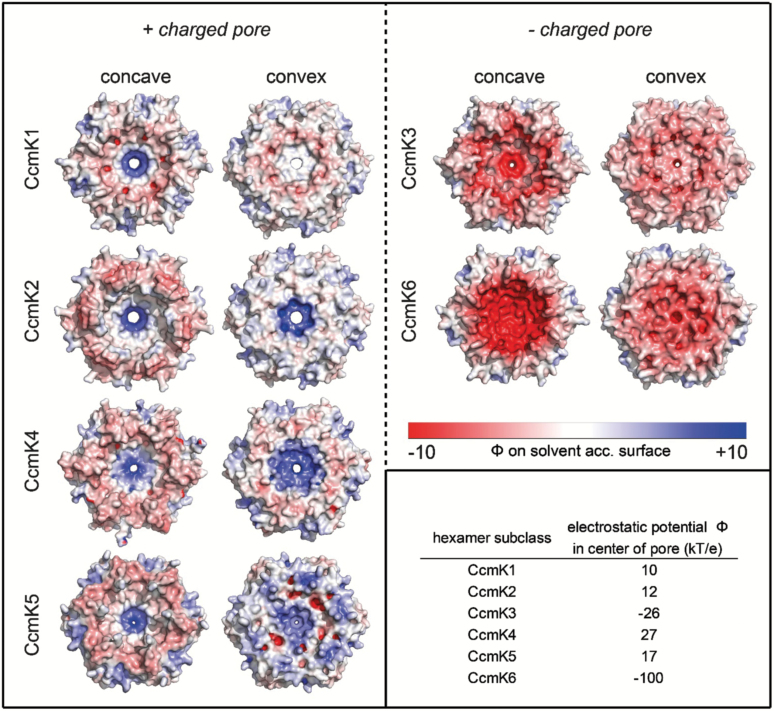
Electrostatic surface potential of CcmK subtypes based on crystal structure (CcmK1, CcmK2, CcmK4) or homology models (CcmK3, CcmK5, CcmK6).

Comparison of the structure-based prediction (homology models for CcmK3, -5, and -6) of the electrostatic surface potential between individual representatives of each CcmK class revealed resemblances between CcmK5 and CcmK1, -2, and -4 (Fig. 3). The positive charge surrounding the pore was contrasted by a slight negative charge in the periphery of the concave side of the hexamer. Residues on the convex side, however, were positively charged. The predicted electrostatic potential in the center of positively charged pores was lowest in CcmK1 and -2 hexamers, slightly increased in CcmK5 hexamer models and was highest in CcmK4 hexamers. In contrast to CcmK5, the predicted surface potential of a CcmK6 hexamer model was uniformly negative with neutral patches only at the inter-hexamer contact sites, making it more like the potential of CcmK3 hexamer models.

**Fig. 4.**
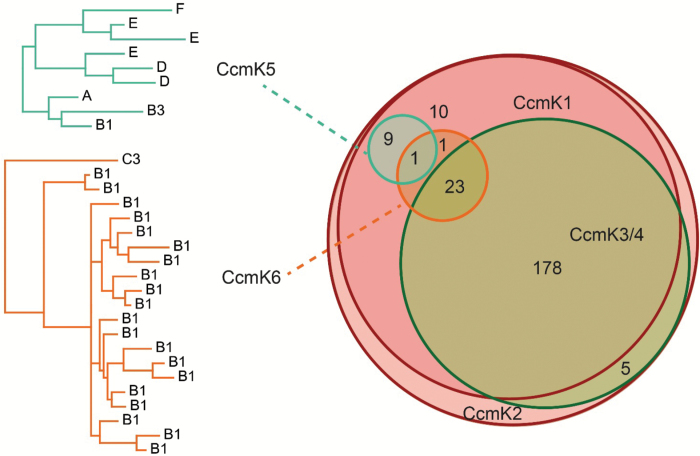
Venn diagram of genome-based occurrences of CcmK proteins. CcmK5 and CcmK6 phylograms are labelled by phylogenetic clades in which they occur.

The co-occurrence of *ccmK* genes was analysed across all sequenced cyanobacteria genomes. At least one copy of the *ccmK1*/*2* class was found in all 227 genomes with β-carboxysomes surveyed in this study (Fig. 4). *ccmK3* and *ccmK4* were found in 206/227 genomes. Notably they always co-occurred. *ccmK5* was found in only 10 genomes; these genomes represented six different systematic clades of the cyanobacterial phylum according to the nomenclature of Shih *et al.* (2013). However, *ccmK5* never co-occurred with *ccmK3* and *ccmK4*. Genes encoding CcmK6 proteins were identified in 25 genomes, almost exclusively within the heterocyst forming B1 clade of cyanobacteria. A chi-squared test was performed on all pairs of *ccmK* genes, to determine if parts of the observed co-occurrence pattern deviated from expected values (Table 1). The observed stringent co-occurrence of *ccmK3* and *ccmK4* deviated from the expected value with high significance. Similarly, the absence of any genome with both a *ccmK5* and a *ccmK3*/*4* gene highly significantly deviated from expectation. No significant deviation involving *ccmK6* was observed.

**Table 1.** Deviation of co-occurrence of CcmK subtypes from the expectation. Values >1 indicate positive correlation Asterisks indicate significant deviation from expected values (**P<0.01, ***P<0.001).

CcmK subtype	Genome coverage (*n*)	Genome coverage (%)	Observed co-occurrences/expected co-occurrences		
			With CcmK3	With CcmK5	With CcmK6
CcmK1/2	227	100.0	1.00	1.00	1.00
CcmK3	206	90.7	—	0.00**	1.02
CcmK4	206	90.7	1.10***	0.00**	1.02
CcmK5	10	4.4	0.00**	—	0.94
CcmK6	25	11.0	1.02	0.94	—

### Beta carboxysome genes form one MCL and one or multiple satellite loci

**Fig. 5.**
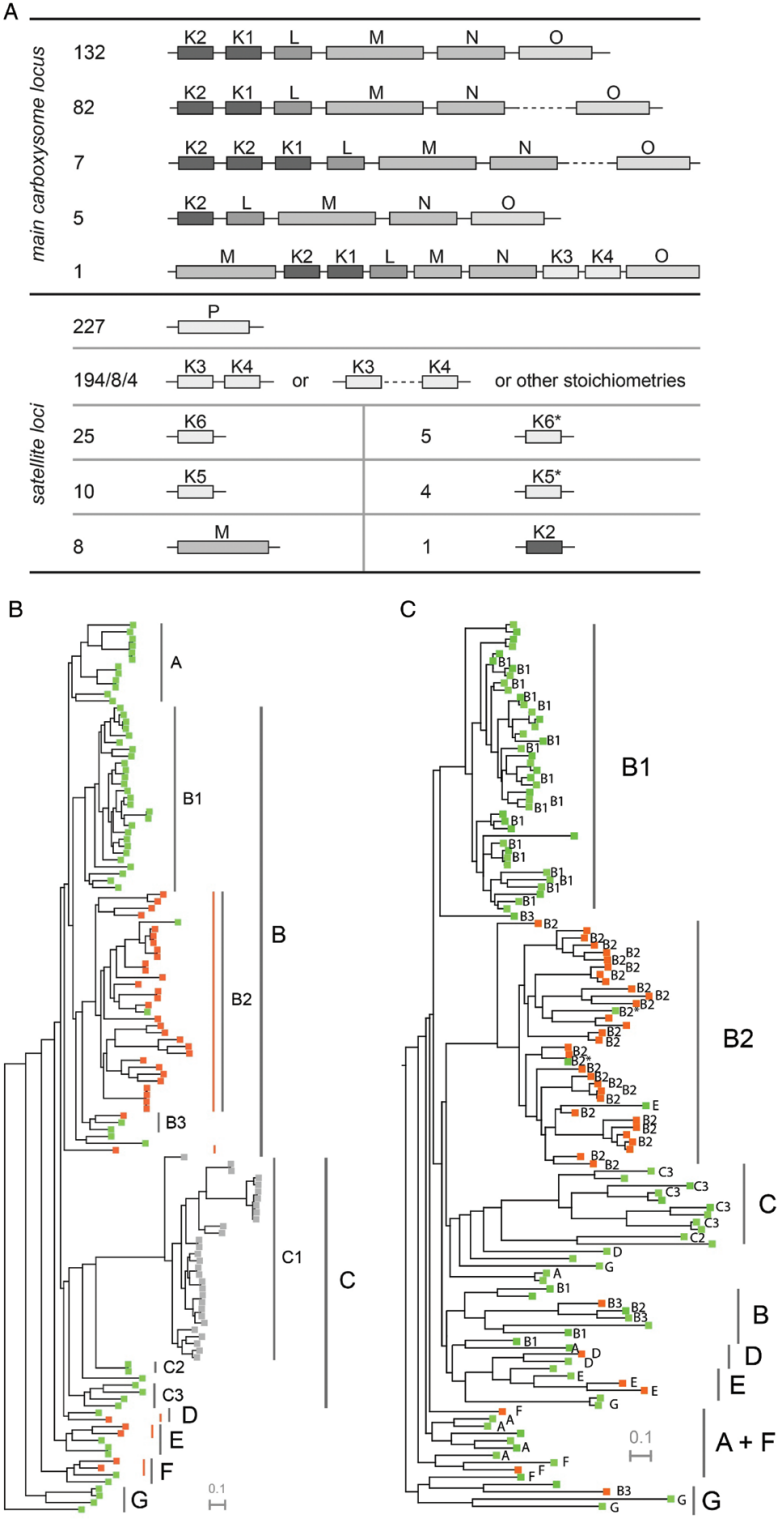
(A) Summary of all loci containing β-carboxysome shell genes in sequenced cyanobacterial genomes. Lengths of genes and distances between genes are not to scale. Supplementary Table S1 lists other stoichiometries of CcmK3 and CcmK4. (B) Species tree of the Phylum Cyanobacteria, color coded by genome position of *ccmO*: main locus *ccmO*: green; satellite *ccmO*: orange; non-β-species: gray. Orange lines indicate phylogenetic groups that arose from five independent *ccmO* relocation events. (C) Phylogram of CcmO amino acid sequences with less than 99% identity, including all beta-cyanobacteria sequenced to-date. Color coding indicates genome position of *ccmO* gene as in (B). Annotation of phylogenetic clades was made for species included in Shih *et al.* (2013). Of species not included, only two species at critical positions of the tree were assigned a clade based on similarity of the rpoC gene (indicated by asterisks).

We next examined the gene organization of the MCL for contents of shell protein genes (Fig. 5A). Each species contains a single MCL that contains one to three *ccmK1*/*2* genes, always followed by *ccmL*, *-M*, *-N*, and in the majority of species, *ccmO*. Only a few of the absolutely conserved MCL genes *ccmK2*, *-K1*, *-L*, *-M* and *-N* had duplicates in satellite loci (see Supplementary Table S1 at *JXB* online). Eight genomes contained a second copy of *ccmM* and only one species, *Fischerella muscicola* PCC 7414, contained a second *ccmK2* gene that was not located in the MCL.

In addition to the MCL, on average 2.5 satellite loci were found in each genome. A satellite locus encoding the CcmP protein was found in all 227 analysed genomes. When *ccmK3* and *ccmK4* were present in a genome, together they formed another satellite locus in 194 species; only eight genomes encoded *ccmK3* and *ccmK4* in separate loci and four genomes had multiple copies of one or both genes. A single species, namely *Chamaesiphon minutus* PCC 6605, had *ccmK3*, *ccmK4* and a second copy of *ccmM*, which lacks the carbonic anhydrase domain, associated with the MCL. The newly identified classes CcmK5 and CcmK6, as well as the subclasses CcmK5* and CcmK6*, were never located close to any other carboxysome gene. There was never more than one copy of either CcmK5 or CcmK6 in a genome. One species, *Cyanothece* sp. PCC 7425, encoded a copy of both CcmK5 and CcmK6.

The genomic position of the BMC-T protein gene *ccmO* was less consistent than that of other carboxysome genes associated with the MCL. In 138 genomes, *ccmO* was the terminal gene in the MCL. However, 89 genomes encoded *ccmO* in a satellite locus that was typically remote from other carboxysome genes. To examine the genomic position of the *ccmO* gene from an evolutionary perspective, species were annotated based on the location of *ccmO* and visualized in a phylogram of the cyanobacterial phylum (species tree, Fig. 5B). The species tree showed a consistent location of *ccmO* in the MCL in the ancestral clade, G, of the phylogram. In the slightly more derived clades, F, E, and D, relocated *ccmO* genes were found in about half the genomes. The highest frequency of genomes with relocated *ccmO* was found in the B clade and particularly in the subclade B2, where only 2 out of 32 genomes located *ccmO* in the MCL. In contrast to this, no relocation event was apparent for any genome in the A clade. In total, at least five independent evolutionary origins of a satellite locus *ccmO* were apparent. A phylogram of CcmO amino acid sequences was built to detect any correlation between divergence of the CcmO primary structure and its genome position (Fig. 5C). The phylogram clustered CcmO proteins from similar phylogenetic clades together, regardless of their location in the respective genome. This arrangement was highly similar to the order seen in the species tree; we conclude there is no difference in primary structure among *ccmO* orthologs that correlates with genome position.

### *The* ccmO *gene is co-regulated with MCL genes only when it is part of the MCL*

To analyse whether localization of shell protein genes to satellite loci was associated with alternative expression of its transcript, Pearson correlations of expression of *ccmK1–4* and *ccmO* with the remaining MCL genes were calculated based on published transcriptome data (Table 2). Two groups of species, representing the MCL-associated or the satellite *ccmO* class, showed concordantly positive correlation between *ccmK1*/*2* and other MCL genes (overall mean: 0.79). Similarly, genomes with *ccmO* in the MCL showed positive correlation values with other MCL genes, and the mean correlation across four species was 0.58. In contrast, species with a relocated *ccmO* gene showed no correlation between the expression of *ccmO* and the remaining MCL genes, and the average mean of correlation was –0.12. The satellite locus genes *ccmK3* and *ccmK4* showed species-dependent variation of correlation with the MCL, with an average mean of 0.42 across all analysed species.

**Table 2.** Correlation of expression of five β-carboxysome shell genes with genes from the main carboxysome locus Analysed datasets are sorted by the genome position of *ccmO* in the respective species.

	Species	Clade	Datasets	Pearson correlation with genes on main locus			Reference
				CcmO	CcmK1/2	CcmK3/4	
*ccmO* satellite	*Synechocystis* 6803	B2	117	–0.07	0.77	0.21	Hernandez-Prieto and Futschik (2012)
	*Synechocystis* 7002	B2	8	–0.09	0.52	–0.03	Ludwig and Bryant (2012*a*)
	*Cyanothece* 51142	B2	12	–0.07	0.84	0.73	Stöckel *et al.* (2008)
	*Microcystis* 7806	B2	33	–0.26	0.92	0.51	Straub *et al.* (2011)
*ccmO* on main locus	*Nostoc* sp. 7120.1	B1	6	0.42	0.91	0.47	Yingping *et al.* (2015)
	*Nostoc* sp. 7120.2	B1	4	0.70	0.90	0.62	Flaherty *et al.* (2011)
	*S. elongatus* 7942*.1*	C2	7	0.51	0.82	0.59	Schwarz *et al.* (2011)
	*S. elongatus* 7942.2	C2	13	0.50	0.92	0.45	Billis *et al.* (2014)
	*Anabaena variabilis* 29413	B1	18	0.90	0.89	0.85	Park *et al.* (2013)
	*Arthrospira platensis* C1	A	6	0.42	0.38	–0.21	Panyakampol *et al.* (2015)

## Discussion

This study inventoried and evaluated the phylogeny of β-carboxysome shell proteins using 227 sequenced β-cyanobacteria genomes, 185 of which were not available before 2013 (Shih *et al.*, 2013). This increase in genomic information has enabled us to identify new classes of β-carboxysome shell proteins and uncover genomic features that underlie the evolution and dynamic regulation of the β-carboxysome shell.

A phylogram of CcmK proteins situates the protein classes CcmK1–4 on three distinct branches (Fig. 1A). The phylogram implies a common origin of CcmK3 and CcmK4 within the CcmK protein family. In contrast, CcmK1 and CcmK2 proteins form a supercluster in the third branch, indicating high similarity between these classes of proteins. Together, CcmK1 and CcmK2 mRNA accounts for the majority of carboxysome-related transcripts in the cyanobacterial model species, *Synechococcus* sp. PCC 6803 (Syn6803) and Syn7942 (Schwarz *et al.*, 2011; Vijayan *et al.*, 2011; Billis *et al.*, 2014); in accord with this, the resemblance of both proteins has been recognized previously in phylogenetic studies that found the proteins on a shared branch (Tanaka *et al.*, 2009; Kinney *et al.*, 2011; Cai *et al.*, 2013, 2015). Low substitution rates within the CcmK1/2 supercluster confirm a particularly high sequence conservation between these two homologs. The highest degree of conservation is found at inter-monomer or inter-hexamer contact sites (Fig. 1D). This is in line with the proposed role of CcmK1 and CcmK2 as the main structural proteins of the carboxysome shell (Kerfeld *et al.*, 2005). An assignment of CcmK1 and CcmK2 homologs based on sequence length within the CcmK1/2 supercluster shows that the phylogram is unable to separate CcmK1 from CcmK2, thus indicating that both protein classes rather belong to a single superclass of proteins (Fig. 1C). The main distinguishing feature between CcmK1 and CcmK2 is the ~10 amino acid long C-terminal extension of CcmK1 (Kerfeld *et al.*, 2005). We hypothesized that this extension leads to sub- or neofunctionalization of CcmK1, which would explain the frequently observed co-existence of CcmK1 and CcmK2 despite their similarity. However, since the model species Syn7942 and four other β-cyanobacteria lack a copy of CcmK1, the presence of its C-terminal extension is not essential for carboxysome structure and function. Furthermore, in a study observing carboxysome assembly from a synthetic, heterologously expressed carboxysome locus, deletion of an extended C-terminus (beyond the Pro90 residue) of both CcmK1 and CcmK2 did not prevent shell assembly, highlighting the limited structural relevance of the C-terminus (Cai *et al.*, 2016). Instead, it might be the genomic proximity of the two *ccmK* genes in the MCL that is advantageous, as suggested by its high degree of conservation (222/227 genomes; Fig. 5A). The sequential arrangement of *ccmK2* and *ccmK1* in the MCL might facilitate protein complex assembly or help balancing shell protein stoichiometry during translation of MCL genes.

In contrast to the highly conserved major shell proteins, CcmK1 and CcmK2, sequence conservation within the classes of the minor shell components CcmK3 and CcmK4 (Schwarz *et al.*, 2011; Vijayan *et al.*, 2011; Billis *et al.*, 2014) is lower, as indicated by long branches within the clusters formed in the phylogram (Fig. 1A). Interestingly, this decrease in conservation involves residues of CcmK3 that are facing the inter-hexamer interface, which suggests a minor role of this protein in higher order oligomerization (Fig. 1D). This hypothesis is further supported by the ubiquitous replacement of an arginine at residue 79 with asparagine in all CcmK3 proteins (Fig. 2A). Arginine 79 is crucial for the stabilization of the inter-hexamer contacts of propanediol-utilizing microcompartments and is conserved across microcompartments and within all CcmK classes with the exception of CcmK3 (Sinha *et al.*, 2014; Pang *et al.*, 2014). Previous studies similarly proposed a minor structural role for CcmK3 and CcmK4 (Rae *et al.*, 2012). Functional redundancy of CcmK3 and CcmK4 was shown for Syn7942, but not for Syn6803, where transposon insertion into *ccmK4* causes premature truncation of the protein, leading to impaired photoautotrophic growth (Zhang *et al.*, 2004; Rae *et al.*, 2012). The physiological functions of CcmK3 and CcmK4, however, remain enigmatic.

The divergence of residues encircling the hypothetical pore (Fig. 2C) and the electrostatic potential of the surface of the CcmK3 model and CcmK4 hexamers (Fig. 3) suggest they may have different functions. However, they always co-occur in genomes and in the vast majority of sequenced genomes they are located in the same locus, suggesting they may functionally depend on each other (Fig. 5A).

In addition to the previously described CcmK protein classes, at least two new CcmK classes were detected (Fig. 1A). While some homologs that apparently did not match the CcmK1–4 classes had been previously noted, the lack of data precluded their assignment to distinct classes and separation of their respective evolutionary paths (Cai *et al.*, 2013, 2015). One of these, CcmK5, is present in species of six phylogenetic clades (Fig. 4). CcmK5 shares a common ancestor with CcmK3 and CcmK4, but the CcmK5 class lies on a basal branch within this cluster (Fig. 1A), indicating that the origin of CcmK5 predates the divergence of CcmK3 and CcmK4. All genomes expressing CcmK5 lack CcmK3 and CcmK4, which is very unlikely to be the result of a random distribution of genes, as indicated by a chi-squared test (Table 2). Based on our findings, we hypothesize that CcmK5 plays a similar functional role as CcmK3/4; presumably the CcmK3/4 locus evolved as the result of a segmental duplication of a single ancestral gene that was more like CcmK5 (Fig. 1A). The similarity of the residues flanking the predicted pore of CcmK4 and CcmK5 and a positive surface potential makes these proteins more likely to function similarly, likely facilitating the transport of small anionic metabolites (Fig. 2C). The swapped pair of residues that are close to the proposed pore of CcmK5 might modify the physical properties of the CcmK5 pore towards a function that CcmK3 and CcmK4 can only achieve together, and thus explain the strict co-occurrence of CcmK3 and CcmK4. Alternatively, the co-expression of CcmK3/4 might provide a functional role that is required only under some environmental conditions. Transcriptomic studies of *Synechococcus* species have shown that the stoichiometry of *ccmK3* and *ccmK4* changes about two-fold in favor of *ccmK3* when high concentrations of CO_2_ are available (Schwarz *et al.*, 2011; Ludwig and Bryant, 2012*b*). This indicates that the relative expression strength of *ccmK3* is increased when carboxysomes turn over CO_2_ rapidly, possibly to adjust the permeability properties of the shell. Interestingly, the combined *ccmK3* and *ccmK4* transcript as compared with the genes for the major shell proteins CcmK1 and CcmK2 decreases under limited availability of CO_2_ or nitrogen (Ludwig and Bryant, 2012*b*). This is consistent with the hypothesis that the CcmK3/4 operon is important for metabolite flux during fast growth conditions. Of the ten genomes lacking CcmK3/4 or CcmK5, five display the very rare K6* gene, and one other genome carries a CcmK6 gene (see Supplementary Table S1); the residues surrounding the pores of the predicted hexamers are atypical among all CcmK proteins (Fig. 2C). The other four genomes are members of the most ancestral cyanobacterial clade. They encode only CcmK1 and CcmK2 proteins. In summary, each cyanobacterial genome (with the exception of the most basal) encodes at least one additional CcmK protein that is distinct from the CcmK1/2 superclass. Based on homology modeling, these paralogs form pores with properties distinct from those of CcmK1/2 hexamers. We suggest these are ancillary shell proteins, not absolutely essential for carboxysome formation. However, our data imply a strong positive selection pressure for their presence; perhaps they are essential in specific environmental conditions.

The second new class of shell protein we identified, CcmK6, is evolutionarily closer to CcmK1 and CcmK2, but is clearly distinct in several ways. The CcmK6 gene is never associated with the MCL (Fig. 5A). The unique sequence of residues that are predicted to surround CcmK6 pores are only remotely similar to CcmK3, and not to any other CcmK class; this further underscores its special role, perhaps as a conduit for a specific metabolite (Fig. 2C). The predominant occurrence of CcmK6 in the heterocyst-forming B1 clade may imply a role under lower ATP and reductant levels or under non-nitrogen limiting conditions (Fig. 4). The strong correlation of CcmK6 proteins with the diazotrophic lifestyle indicates that encoding additional shell genes in satellite loci may reflect adaptation of the carboxysome to a specialized physiology, but studies of species encoding a CcmK6 protein are required to test this hypothesis.

Including the newly identified BMC-H classes, the number of homologs of carboxysome shell proteins with a single Pfam00936 domain can be as high as six per genome (see Supplementary Table S1). A comparison with other types of channel proteins is instructive in understanding this multiplicity. For example, the well-studied pores of mammalian ion channels also display a narrow pore surrounded by a conserved array of residues. Mutations in a single residue can change substrate specificity of a potassium transporter for the alternative substrates ammonium and rubidium, or even convert a sodium channel into a calcium channel (Yool and Schwarz, 1991; Heinemann *et al.*, 1992). Similarly, the specificity of potassium channels is based on a small increase in their pore diameter as compared with sodium channels, which makes the passage of the 0.4 Å smaller sodium ion more than 10^5^ times less likely than potassium ion passage (Doyle *et al.*, 1998). Single residue alterations observed among different CcmK proteins, especially in the pore region, could likewise account for substantial differences in selectivity as a result of changes in diameter or charge (Fig. 3). Consequently, a high number of subfunctionalized CcmK proteins could regulate metabolite flux across the carboxysome shell more specifically and dynamically, if their expression can be regulated independently.

Prokaryotic genes are frequently organized in operons to co-regulate their expression, while genes that are not part of an operon can be expressed individually (Jacob and Monod, 1961). Previous studies of cyanobacterial gene expression found that *ccmK* and *ccmL*, as well as the core proteins *ccmM* and *ccmN*, are co-regulated in an MCL as an operon (Price *et al.*, 1993; Rae *et al.*, 2013; Billis *et al.*, 2014). The present study confirms the presence of a main operon-like locus, containing *CcmK2*/*K1*/*L*/*M*/*N* in 222/227 β-cyanobacterial genomes (Fig. 5A). However, the term locus is preferred over the term operon here, since some facultative members of the MCL, namely *ccmO*, *rbcL*, and *rbcS*, are less strictly co-regulated with the core genes (Billis *et al.*, 2014). Only five β-cyanobacterial genomes lack the *ccmK1* gene, one of them the model species Syn7942, which makes *ccmK2*/*L*/*M*/*N* the minimum genetic unit of the MCL. The high average Pearson correlation between expression of *ccmK1* and *ccmK2* and other MCL genes in eight cyanobacterial species confirms a transcriptional co-regulation of MCL genes (Table 2).

In addition to the MCL, each β-cyanobacterial genome contains at least one satellite locus with carboxysome genes, which implies that this dispersal of genes is essential (see Supplementary Table S1). The satellite BMC-H genes *ccmK3* and *ccmK4* show only moderate co-regulation with MCL genes, as was previously demonstrated in the model species Syn7942 and Syn6803 (Table 2) (Billis *et al.*, 2014). For another satellite locus BMC gene, *ccmP*, the co-regulation with MCL genes was shown to depend on environmental conditions (Cai *et al.*, 2012). This dynamic expression of satellite locus carboxysome shell genes potentially alters the protein composition of the shell, which may affect the permeability of a carboxysome, adapting it to environmental conditions. The dispersal of carboxysome genes across genomes was apparently an ancient adaptation to fluctuating resources in β-cyanobacteria, as the conserved satellite location of *ccmP* and *ccmK3–6* implies.

Satellite copies of genes of the MCL, namely *ccmK2*, *ccmK1*, and the carbonic anhydrase *ccmM*, were identified in few genomes (Fig. 5A). The single satellite *ccmK2* found in *Fischerella muscicola* PCC 7414 may pose a unique adaptation to its environment. The rarity of satellite copies of *ccmK1* or *ccmK2* implies that their exclusive location to the MCL, as part of an operon with *ccmL*, *-M* and *-N*, is advantageous for most cyanobacteria, perhaps reflecting the importance of concerted control of the stoichiometry of carboxysome proteins for assembly. For *ccmM*, which is a fusion of a γ-carbonic anhydrase domain and three to five RubisCO small subunit-like domains (SSLDs), smaller satellite duplicates with a single SSLD were previously described in the *Leptolyngbia* and *Acaryochloris* family (Long *et al.*, 2011). The additional satellite *ccmM*s we identified in *Microcoleus* and *Rubidibacter* display the same reduction in number of SSLDs. A single SSLD fused to RubisCO activase (Rca) was proposed to recruit Rca to the vicinity of its target, RubisCO (Zarzycki *et al.*, 2013). The single SSLD in secondary CcmM proteins may fulfil a similar function. Especially *Rubidibacter*, which lacks a copy of the ancillary carboxysomal carbonic anhydrase, CcaA, may require the extra activity contributed by the secondary CcmM (see Supplementary Table S1). In the unique MCL of *Chamaesiphon*, the second CcmM consists only of three SSLDs and takes the place of *ccmM* in the MCL, while the longer (carbonic anhydrase domain and three SSLDs) *ccmM* is in an uncommon position, upstream of *ccmK2*. Analogously, the model species Syn7942, which contains only a single *ccmM* gene, expresses two forms of the protein containing either the complete sequence or just the three SSLDs (Price *et al.*, 1998; Long *et al.*, 2005). The separation of this ‘long’ and ‘short’ form of CcmM in *Chamaesiphon* to two genes may help independent regulation of their expression; further studies of gene expression in *Chamaesiphon*, however, are necessary to test this hypothesis. None of the analysed genomes contains a second copy of *ccmL* or *ccmN* and both are exclusively located in the MCL. Thus, co-regulated expression of the minimal genetic unit of the MCL is a given in nearly all β-cyanobacteria.

The BMC-T (tandem Pfam 00936 domain) protein CcmO is the only shell protein in β-carboxysomes that can be encoded as either part of the MCL or as a satellite gene in significant numbers of genomes. The ancestral state of the *ccmO* gene is in the MCL, as this is the observed location in the most ancestral phylogenetic clade G of cyanobacteria (Fig. 5B) (Shih *et al.*, 2013). Even though *ccmO* does not meet the criteria for being part of the main carboxysome operon, its frequent association with the MCL suggests a biological relevance for this genome arrangement. However, at least five independent evolutionary origins of separation of *ccmO* from the MCL implies there is a selective pressure for relocation, or low pressure to retain *ccmO* in the MCL. Given that many species retain *ccmO* in the MCL, this evolutionary force seems to apply only in specific environments. Because genomes with satellite *ccmO* genes do not belong to a single morphological subtype or inhabit similar ecological niches (see Supplementary Table S1) (Shih *et al.*, 2013), diverse environmental conditions appear to promote relocation of *ccmO*.

From the branching pattern of a phylogram analysing MCL- and satellite-*ccmO* sequences, no apparent difference exists between the two groups of proteins (Fig. 5C). Furthermore, CcmO expressed from both locus types has been shown to be associated with heterologously expressed carboxysome shell structures (Lin *et al.*, 2014; Cai *et al.*, 2016). However, the regulation of its gene expression varied dramatically in conjunction with its position within or outside of the MCL (Table 2). The consistent, moderately positive correlation between the expression of *ccmO* and other MCL genes when *ccmO* is part of the MCL highlights the character of this locus as a regulatory unit. In contrast, there is an absence of co-regulation between satellite *ccmO* and MCL genes; this is consistently observed in four exemplary, ecologically diverse species; the represented habitats range from marine sediment (*Synechocystis* sp. PCC 7002) to a toxin-producing cosmopolitan that can grow in freshwater, soil and even conditions mimicking the human gut (*Microcystis* sp. PCC 7806) (Stefanelli *et al.*, 2014). For another facultative MCL gene, *rbcL*, a similar divergence could not be observed (see Supplementary Table S2). Instead, expression of *rbcL* is mostly correlated with MCL genes even when *rbcL* is not associated with the MCL. The extreme isolation of the regulatory pattern of *ccmO* is thus unique among all carboxysome shell genes and is not paralleled by a luminal enzyme. Transcriptomic studies of species with a relocated *ccmO* gene from clade D, E and F have not been published to date, but will be valuable for a more comprehensive understanding of the dynamic regulation of *ccmO*. Based on the results presented here, we conclude that the evolutionary force driving the relocation of *ccmO* in β-cyanobacteria is the ability to regulate its expression independently. Thus, the relocation of *ccmO* to satellite loci reveals the potential to increase plasticity of expression of β-carboxysomes.

The structural building blocks of the shell, BMC-H, BMC-T and BMC-P proteins, are conserved among functionally distinct BMC types (Axen *et al.*, 2014; Kerfeld and Erbilgin, 2015). Some sequence motifs, like the residues encircling the pore of CcmK1/2, (KIGSG, Fig. 2C), are also found among shell proteins of at least four types of catabolic microcompartments (Bobik *et al.*, 1999; Zarzycki *et al.*, 2015). Despite this conservation, the vast majority of catabolic BMCs and carboxysomes (anabolic BMCs) contain multiple BMC-H paralogs with diversity among the residues encircling the pore, which leads to unique structural constraints for metabolite transport (Kerfeld *et al.*, 2005; Crowley *et al.*, 2008). These evolutionary parallels imply that acquisition, associated with subfunctionalization, of multiple BMC-H proteins may be a common way to adaptively adjust the permeability of the BMC shell (Kerfeld *et al.*, 2010; Kinney *et al.*, 2011; Axen *et al.*, 2014; Kerfeld and Erbilgin, 2015; Zarzycki *et al.*, 2015).

The dynamic regulation of shell permeability, enabled by distribution of BMC proteins to multiple loci, however, appears to be a unique adaptation to the functional constraints of β-carboxysomes. In contrast, isolated satellite loci have never been observed in α-cyanobacterial genomes; indeed, they can encode for as few as one BMC-H protein (Rocap *et al.*, 2003; Roberts *et al.*, 2012; Axen *et al.*, 2014). However, the expression of the α-cyanobacterial BMC-T homolog CsoS1D is not stringently correlated with the expression of other α-carboxysome genes and it is found separated from the MCL by a single non-carboxysome gene in all studied α-cyanobacteria (Klein *et al.*, 2009; Roberts *et al.*, 2012). It is thus possible that both types of carboxysomes have the same requirement for plasticity to some extent, but took different evolutionary paths to cope with it. The higher degree of positional distribution of carboxysome genes across the genome in β-cyanobacteria might explain their potential to occupy very diverse niches, while α-cyanobacteria are mainly confined to open ocean ecosystems (Badger *et al.*, 2006).

The genomes encoding non-cyanobacterial α-carboxysomes (found in chemoautotrophs) and catabolic BMCs likewise seldom encode satellite BMC loci; satellite loci are found in only 10% of these genomes (Axen *et al.*, 2014). Expression of catabolic BMCs is dependent on the presence of certain nutrients, unlike β-carboxysome genes, which are constitutively expressed (Bobik *et al.*, 1992; Rondon and Escalante-Semerena, 1992; Roof and Roth, 1992; Kusian and Bowien, 1997; Rae *et al.*, 2013; Erbilgin *et al.*, 2014; Kerfeld and Erbilgin, 2015). Facultative expression of genes for shell paralogs encoded in satellite loci may reflect the particular needs of ecophysiologically diverse cyanobacteria to alter the composition of the β-carboxysome shell in response to the environment. In contrast, the all-or-none expression model for a single locus of BMC genes (e.g. for catabolic BMCs or the α-carboxysomes of chemoautotrophs) is more likely to evolve where the availability of the substrate, the trigger for formation of the BMC, is either on (available) or off (not available). Carbon fixation by β-carboxysomes, however, is both essential and must be responsive to fluctuating substrate availability, which makes dynamic regulation of its shell proteins, which serve as the interface to the rest of cyanobacterial metabolism, preferable. This is facilitated by location of carboxysome shell protein genes to satellite loci.

### Conclusion and future prospects

Based on the comprehensive analysis of carboxysome shell genes presented here, we conclude that genome rearrangement, in the form of acquisition of BMC-H paralogs and relocation of *ccmO* (a BMC-T protein), is an essential and frequently observed mode of adaptive evolution of the β-carboxysome structural genes. The expanded set of paralogs, in conjunction with the increased variability in numbers and classes expressed, presumably increases the plasticity of the microcompartment shell. This could reflect its adaptation to distinctive environmental and or physiological conditions. Other BMC types do not require a similar level of regulation, as evident by the localization of all BMC genes in a single operon (Axen *et al.*, 2014). For future work with genes for carboxysome shell proteins, especially their heterologous expression in plant hosts to enhance carbon fixation, it will thus be essential to understand this demand for plasticity. Analysis of the regulatory patterns of carboxysome shell genes will be a vital next step on the way towards functional application of carboxysomes in crop plants and will be generally applicable for developing BMCs for applications in synthetic biology (Hanson *et al.*, 2016; Cai *et al.*, 2016; Gonzalez-Esquer *et al.*, 2016*a*).

## Supplementary data

Supplementary data are available at *JXB* online.

Table S1. Annotation of all β-cyanobacterial genomes with information about carboxysome shell genes from bioinformatic analysis that is relevant to this study.

Table S2. Correlation of expression of the *rbcL* gene with genes from the main carboxysome locus. Analysed datasets are sorted by the genome position of *rbcL* in the respective species.

Table S3. IMG gene IDs of amino acid sequences used to create initial HMMs.

## Supplementary Material

supplementary_table_S1

supplementary_table_S2

supplementary_table_S3

## Abbreviations:

BMC: bacterial microcompartment
CCM: carbon concentrating mechanism
HMM: hidden Markov model
MCL: main carboxysome locus
PCC: Pasteur culture collection
Rca: ribulose-1,5-bisphosphate carboxylase oxygenase activase
RubisCO: ribulose-1,5-bisphosphate carboxylase oxygenase
SSLD: RubisCO-small-subunit-like domain.
